# Video-Assisted Learning for Oral Health Knowledge in Children: A Pre-post Intervention Study

**DOI:** 10.7759/cureus.103638

**Published:** 2026-02-15

**Authors:** Beatriz Miguel, José Pedro Mendes, Giulia Ober

**Affiliations:** 1 Family Medicine, Family Health Unit Oriente, Saint Joseph's Local Health Unit, Lisbon, PRT

**Keywords:** health knowledge, oral health, oral health behaviours, school children, video assisted learning

## Abstract

Introduction

Tooth decay is a common childhood disease. There is growing recognition of the value of community-based, personalised, and digitally driven interventions in enhancing oral healthcare.

Methodology

One hundred and fifty-five students from two schools with the highest “decayed, missing, and filled teeth (DMFT)” indices in Lisbon were selected. The intervention consisted of a four-week program featuring educational videos on oral health (tooth structure, hygiene, diet, and diseases), with pre-, post-intervention, and three-month follow-up assessments. The sessions were facilitated by family medicine resident doctors, oral health hygienists, and teachers. Children who did not complete the program were excluded. Guardian-informed consent was obtained. The data was anonymised. Statistical analysis included repeated-measures analysis of variance (ANOVA) and McNemar tests.

Results

A hundred and eight children were included (mean age 9.24 ± 0.75 years, range 8 - 11). After the intervention, the proportions of children brushing two to three times/day and flossing daily increased (from 77.8% to 85.2% and from 42.6% to 49.1%, respectively). From baseline, the mean dental health knowledge score improved by 1.77 points after the intervention [9.47 to 11.24/13, 95% confidence interval (CI) 2.21 - 1.32, p < 0.01] and by 0.52 points at follow-up (9.47 to 9.99, 95%CI 0.95 - 0.08, p = 0.014).

Conclusion

This video-based intervention enhanced student engagement with oral health content while providing valuable insights into the effectiveness of digital education in promoting better oral health knowledge among children.

## Introduction

Oral health is a key determinant of overall well-being and quality of life, strongly affecting development and self-esteem across all stages of life [1]. However, dental caries remains one of the most prevalent chronic diseases in childhood. Despite their widespread prevalence, the majority of oral diseases are preventable through accessible self-care practices, public health interventions, and the implementation of cost-effective, population-wide strategies that address the broader social, economic, and political determinants of health. In response to this global burden, the World Health Organisation (WHO) developed a strategic plan in 2022, which aimed for Universal Oral Health Coverage by 2030, targeting 80% of the global population to have access to essential oral health care services and also aiming to have a relative reduction of 10% in the combined global prevalence of the main oral diseases and conditions over the life course [2-3]. WHO also developed a set of guiding principles under the Global Strategy on Oral Health. These principles advocate for a public health approach to oral health, the integration of oral health into primary health care, and the provision of people-centred programs. This strategy emphasizes the importance of delivering tailored interventions across the life course and harnessing the potential of digital technologies to strengthen oral health awareness. It also emphasises the need for coordinated efforts involving families, schools, and the community, while advocating for personalised, life-course interventions and the integration of digital technologies to improve oral healthcare delivery [2-4].

Consistent with this view, Portugal launched the National Programme for Oral Health Promotion (Programa Nacional de Promoção da Saúde Oral - PNPSO) in 2023, intending to reduce the burden of oral diseases nationwide [5]. This programme advocates the creation of community-based health-promoting activities, targeting younger individuals, and easier access to dental care through the utilisation of a dental voucher as part of a national oral health programme, which provides children with free access to preventive and restorative dental care. A primary objective was to increase the proportion of cavity-free six-year-olds to 80%, compared to 54% recorded in 2015, and to reduce the decayed, missing, and filled teeth (DMFT) index at 12 years of age to 1.15 by 2025, relative to a baseline of 1.18 in 2015 [5]. To monitor the main oral health indicators, the Third National Study for the Prevalence of Oral Disease was conducted in 2015. This study adapted the International Caries Detection & Assessment System (ICDAS) to better assess Portugal's oral health status. According to this study, only 54.8% of six-year-olds and 53.0% of 12-year-olds were caries-free. Additionally, just 53.0% of six-year-olds and 69.9% of 12-year-olds reported brushing their teeth more than twice a day, while the majority (68.3%) did not use dental floss [6].

In parallel with an up-to-date perception of childhood dental health (either by DMFT index or caries-free percentage), a correct assessment of oral health knowledge [7], perception, and behaviours is also fundamental to developing strategies towards better oral healthcare [8]. Evidence shows that, in Portugal, basic dental care knowledge in school children might be lacking [9]. On the other hand, as previously stated, oral health behaviours amongst Portuguese children might be far below desirable levels [6, 9, 10]. Schools provide an opportunistic and adequate environment to deliver and optimize information on oral health topics [11]. Furthermore, oral health interventions based on social cognitive theory, such as video content or gamification, are effective in delivering information while promoting engagement [11, 12].

During the 2022-2023 academic year, the Oral Health Team of São José's Local Health Unit (LHU) in Lisbon, as part of the National Program for Oral Health Promotion, aimed to assess the oral health status of children between the ages of four and 13, and to evaluate the prevalence of dental caries in 100 kindergartens and schools, including public, private, and social network institutions. This data was presented in a local Public Health meeting and in an internal report in October 2023. The results showed a DMFT index of 1.23 at seven years of age, higher than at ages six, five, and four (respectively, 1.07, 0.79, and 0.38), indicating an age-related upward trend in the study population. These findings highlighted the urgent need for targeted local interventions.

In alignment with the WHO Global Strategy on Oral Health and in response to national and local data, our project was developed to improve oral health promotion in school programmes, children’s knowledge and behaviours regarding oral health, and to reinforce the connection between educational institutions and the health care system. The intervention aimed to improve students’ knowledge and to promote behavioural change through an educational approach centred on video-based learning. Therefore, the objectives of this intervention project were to assess the impact of a video-based educational intervention on oral health knowledge and behaviour of children in third and fourth grades from two schools with the highest DMFT indexes in the Saint Joseph's Local Health Unit region in Lisbon.

## Materials and methods

Participants

A total of 155 students from two primary schools within the area of Saint Joseph's Local Health Unit in Lisbon were enrolled in this pre-post intervention study. These schools were selected based on their notably high DMFT indices (3.52 and 1.84, respectively). Assuming a baseline toothbrushing rate of 70% (according to national estimates) [6, 8] and expecting an increase to 90% following the intervention, the required sample size was calculated using McNemar’s test for paired proportions, with a two-tailed significance level of 0.05 and a statistical power of 80%. This calculation indicated a minimum sample size of 40 participants to detect a meaningful change. Inclusion criteria comprised children enrolled in third and fourth grades with sufficient oral and written proficiency in Portuguese. Children with language barriers, defined as the inability to speak, read, or write Portuguese, were excluded from the study. Additionally, participants who did not complete the full intervention due to school transfer, withdrawal, or absence from one or more sessions were excluded from the final analysis.

Instruments

The scripts for four educational videos (Appendices 1, 2, 3, 4) were developed in collaboration with oral health hygienists and primary school teachers, and subsequently produced with the support of a professional graphic designer. Six questionnaires (Appendices 5, 6, 7, 8, 9) were also designed to evaluate the intervention’s impact. All materials were reviewed and validated by oral health specialists and primary school teachers to ensure scientific rigor, accuracy, and relevance to the educational objectives. Each video focused on an oral health topic: tooth structure (Appendix 1), dietary concerns (Appendix 2), oral hygiene (Appendix 3), and common oral diseases (Appendix 4). The first video, with a duration of three minutes and 26 seconds, covered the different types of teeth and their development from childhood to adulthood. It detailed the functions of various teeth and emphasized their roles in mastication, speech articulation, respiratory support, facial aesthetics, and overall systemic health. The anatomical structure was explained, highlighting the crown as the visible portion and the root beneath the gingiva. The second video, lasting two minutes and 51 seconds, addressed dietary concerns by identifying foods beneficial to dental health and providing recommendations to limit cariogenic food intake, as well as explaining the process of dental plaque formation. The third video, with a duration of three minutes and two seconds, focused on oral hygiene practices, covering the recommended frequency for toothbrush replacement, effective brushing techniques, and proper dental floss use. The final video, lasting four minutes and 24 seconds, reviewed common dental and periodontal diseases, described the roles of oral health professionals, and advised on when to seek dental care. It also illustrated the aetiologies of dental caries, periodontal disease, and halitosis, reinforcing the role of dental plaque in these conditions. All videos used an age-appropriate language.

Intervention

During the 2024/2025 academic year, the oral health education program was delivered across five structured sessions over four weeks, incorporating age-appropriate educational videos, as outlined in Table 1.

**Table 1:** Sequence of intervention sessions, indicating the facilitators involved, the timing of video presentations, and when the questionnaires were administered (before or after each video)

Intervention Sessions	Session	Applied Materials Questionnaires and Videos	Model	Number of questions	Facilitators	Questionnaires Applied
	1^st^ Session 30min	Diagnostic questionnaire (Appendix 6, 7, 8, 9)	1	13	Investigators, Oral Hygienists, Teachers	Before the video
		Behaviour characterization questionnaire (Appendix 5)		4		
		Visualization of Video 1 (Appendix 1)	2	3		After the video
		Specific questionnaire for Video 1 (Appendix 6)				
	2^nd^ Session 30min	Visualization of Video 2 (Appendix 2)	3	4	Teachers	
		Specific questionnaire for Video 2 (Appendix 7)				
	3^rd^ Session 30min	Visualization of Video 3 (Appendix 3)	4	3		
		Specific questionnaire for Video 3 (Appendix 8)				
	4^th^ Session 30min	Visualization of Video 4 (Appendix 4)	5	3		
		Specific questionnaire for Video 4 (Appendix 9)				
Three months after the Intervention	5^th^ Session 30min	Final Questionnaire (Appendix 6, 7, 8, 9)	6	13	Investigators, Oral Hygienists, Teachers	Without the video
		Behaviour characterization questionnaire (Appendix 5)		4		

Procedure

All participating schools received an identical, standardized intervention, consisting of five structured sessions. The intervention was not modified during the course of the study. At the start of the academic year, a preparatory training session was delivered to all participating teachers to ensure standardized implementation. This training covered both the procedural aspects of the intervention and the technical steps for delivering digital video content. It included a presentation, the administration of questionnaires, the viewing of educational videos, and a question-and-answer session to clarify any doubts through a structured and interactive approach. To ensure fidelity of the intervention across different schools, protocols were developed to maintain uniformity in its implementation, particularly in sessions delivered solely by teachers who were not involved in the development of the project. These protocols included detailed guidelines and structured materials (questionnaires and video content), as well as step-by-step instructions on how to conduct each part of the intervention. This helped standardize delivery and minimize variability between facilitators. No formal assessment of adherence was conducted, but the use of these standardized materials served as a strategy to promote consistency. The intervention was delivered fully according to the pre-established protocol and timeline.

The sessions were scheduled weekly during November and December 2024. The intervention took place during school hours, within the classroom setting, and was always delivered by trained facilitators following a standardized protocol to ensure consistency. The first and fifth sessions were facilitated by the intervention team, composed of three family medicine interns and dental hygienists, together with the class teacher, whereas the second, third, and fourth sessions were conducted independently by the respective classroom teachers. During this first session, any queries from teachers regarding intervention procedures were addressed directly by the medical interns and dental hygienist. Following each of the second, third, and fourth sessions, teachers were instructed to distribute and collect student questionnaires immediately after the video presentations. In the post-intervention session (Session 5), the correct answers to the questionnaires were reviewed interactively with students and teachers, facilitated by medical interns and the dental hygienist. Post-intervention activities took place in March 2025. All sessions were delivered face-to-face in classroom settings, with the intervention administered to each class as a whole group.

During the first session, students completed a diagnostic questionnaire consisting of two sections: one assessing baseline oral health knowledge (Appendix 6, 7, 8, 9) and another evaluating oral health-related behaviours (Appendix 5). The knowledge section contained 13 questions, identical to those administered following each subsequent video session. The behaviours section included four items focused on daily oral hygiene practices. Each oral health topic - tooth anatomy (Appendix 6), dietary considerations (Appendix 7), toothbrushing techniques (Appendix 8), and common oral pathologies (Appendix 9) - was addressed through a dedicated video presented in Sessions one, two, three, and four, respectively. Immediately after each session, students completed a topic-specific questionnaire designed to assess comprehension of the material covered. These questionnaires contained three, four, three, and three questions, respectively, as detailed in Table 2. Three months after the conclusion of the fourth session, a final session (session five) was conducted. In this session, students completed a comprehensive questionnaire identical to the initial diagnostic tool, enabling evaluation of long-term knowledge retention. No video content or reinforcement activities were provided between sessions four and five, ensuring that the assessment reflected sustained knowledge from the intervention. 

**Table 2:** Questions on knowledge, according to specific topics (tooth structure, diet, oral hygiene, and common oral diseases) The correct answers are highlighted in bold.

Topics	Question 1	Question 2	Question 3	Question 4
Tooth structure	How many permanent teeth do we have?	In which parts are the teeth divided?	Which one of the following helps keep the enamel strong?	Not applicable
	32	Crown, gum, and tooth base	Sugar	Not applicable
	40	Head, gum, and tooth truncus	Fluoride	Not applicable
	20	Crown and tooth root		Not applicable
Diet	Which of the following damages your teeth?	When can I eat sugary food?	To keep your teeth healthy, what must you do every night before going to bed?	What increases your dental plaque?
	Gums and cookies	Never, it is forbidden	Eat dessert	Not brushing my teeth twice daily
	Apples and steaks	On holidays or party days	Brush my teeth	Not eating sugary food
	Carrots and broccoli	Whenever I feel like	Drink juice	Not applicable
Oral hygiene	At a minimum, how many times a day should we brush our teeth?	My toothpaste should have…?	At a minimum, how many times a day should we floss our teeth?	Not applicable
	One time	Calcium	One time	Not applicable
	Two times	Fluoride	Two times	Not applicable
	Four times	Phosphorus	Three times	Not applicable
			Four times	Not applicable
Common oral diseases	Which of the following are the most common problems of teeth and gums?	Who visits you at school to observe your teeth and teach you how you should take care of your teeth?	How can you keep your teeth healthy?	Not applicable
	Caries and gingivitis	Dentist	Eat many times	Not applicable
	Unaligned teeth	Family doctor	Brush my teeth	Not applicable
	Broken teeth	Oral hygienist	Brush and floss my teeth, and eat healthy	Not applicable

Parental informed consent was obtained at the beginning of the academic year during a parents’ meeting held at each school. The study protocol received ethical approval from the Ethics Committee of Saint Joseph's Local Health Unit 1546/2024. To preserve confidentiality, all data were anonymised through the allocation of a unique six-digit code to each student. 

Data analysis

Demographic variables assessed were sex, age, and school grade. Concerning behaviours, the following variables were evaluated: daily tooth brushing (yes/no), how many times the daily brushing was performed (1, 2, 3, or 4), daily tooth flossing (yes/no), and how many times the student had attended the oral hygienist/dentist the previous year (open question). Regarding knowledge, we employed questions on tooth structure, diet, oral hygiene, and common oral diseases, with only one right answer for each question. Each right answer was given a point. Knowledge was measured as a percentage of right answers for each topic and for the total number of questions. Statistical analyses were performed using repeated-measures analysis of variance (ANOVA) to evaluate changes in oral health knowledge and behaviours over time. McNemar’s test was applied to compare paired pre- and post-intervention categorical responses. All analyses were conducted using IBM SPSS Statistics version 29.0 software (IBM Corporation, Armonk, New York), with the level of statistical significance set at p < 0.05.

## Results

Sample characterization

Initially, 155 children from both schools were enrolled in the study, but only 108 were included in the analysis due to incomplete answers. Most students were male (60.2%, N = 65), with a mean age of 9.24 ± 0.75 years (range 8 - 11). The majority of students were fourth graders (60.2%, N = 65).

Behaviours

Regarding the student’s baseline behaviour, although most students brushed their teeth every day at least once (93.5%, N = 101), less than half flossed their teeth (42.6%, N = 46). When asked about the number of times they brushed their teeth, 77.8% (N = 84) replied two or three times/day. Regarding dentist/oral hygienist access, only nine children replied that they did not attend an appointment in the previous year. At baseline, no statistically significant relation between age, sex, or school years and children’s oral health behaviours was found.

Three months after the intervention, a McNemar analysis found no difference in the number of children who brushed their teeth daily (93.5%, N = 101; X^2^ = 31.694, p = 1) or who went to the dentist/oral hygienist in the previous year (7.4%, N = 8; X^2^ = 22.839, p = 0.344). However, an increase in the number of students who floss their teeth was observed (+ 6.5%; 49.1%, N = 53), as well as in the number of children who brushed their teeth two to three times/day (+7.4%; 85.2%, N = 92), although none were statistically significant (X^2^ = 23.396, p = 0.265 and X^2^ = 12.583, p = 0.134, respectively). The number needed to treat to get one more child to brush their teeth a correct number of times was 15. The full behaviour characterization is shown in Table 3.

**Table 3:** Full behaviour’s characterization A comparison between behaviours at baseline and after three months was performed using a McNemar test. *This was an open question: the expected answers were two to three times a day. **This was an open question: the expected answer was at least once.

Questions	Answer	Baseline		3 months		X^2^	p-value
		N	%	(N)	(%)		
Do you brush your teeth every day?	Yes	101	93.5%	101	93.5%	31.694	1
	No	7	6.5%	7	6.5%		
How many times do you brush your teeth?^*^	1	12	11.1%	11	10.2%	12.583	0.134
	2	60	55.6%	64	59.3%		
	3	24	22.2%	28	25.9%		
	4	12	11.1%	0	0%		
	No answer	0	0%	5	4.6%		
Do you floss your teeth daily?	Yes	46	42.6%	53	49.1%	23.396	0.265
	No	62	57.4%	55	50.9%		
How many times did you attend the oral hygienist/dentist last year?^**^	0	9	8.3%	12	11.1%	22.839	0.344
	1	24	22.2%	15	13.9%		
	2	40	37.0%	34	31.5%		
	3	21	19.4%	25	23.1%		
	4	7	6.5%	8	7.4%		
	5	2	1.9%	4	3.7%		
	6	2	1.9%	2	1.9%		
	7	1	0.9%	8	7.4%		
	No answer	2	1.9%	8	7.4%		

Knowledge

**Figure 1:**
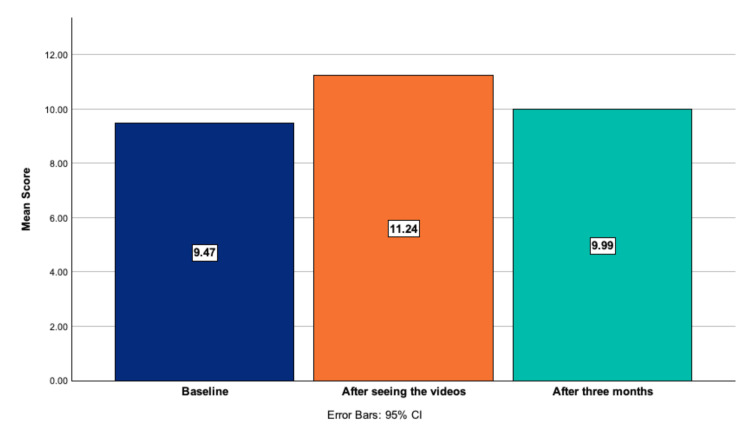
Evolution of the knowledge score at the three timepoints

The mean baseline knowledge score (timepoint 1) was 9.47 ± 1.69 out of 13 (72.8%, score range 13 - 5). Third graders scored a mean of 9.70 ± 1.73 (74.6%, range 13 - 5) and fourth graders scored a mean of 9.32 ± 1.67 (71.7%, range 13 - 5). Immediately after seeing the videos (timepoint 2), the mean knowledge score was increased to 11.24 ± 1.47 out of 13 (86.5%, range 13 - 6). Three months later (timepoint 3), without seeing the videos, the mean knowledge score was 9.99 ± 1.68 out of 13 (76.8%), as shown in Figure 1 and Table 4.

**Table 4:** Knowledge mean scores at the three timepoints, according to each specific topic A repeated-measures ANOVA with a Greenhouse-Geisser correction indicated that the scores differed significantly over time (F(1.857, 198.671) = 57.512, p < 0.01). A post hoc analysis with a Bonferroni adjustment showed that the knowledge score significantly increased from baseline to immediately after the intervention (+1.77 points, p < 0.001) and from baseline to three months after the intervention (+0.52 points, p = 0.014). *Statistically significant (p < 0.05). Abbreviations: SD - standard deviation; CI - confidence interval; TP – timepoint.

	Mean Score			95% CI	p-value
Topics	Baseline	After the videos	3 months		
	(x̄ ± SD, %)	(x̄ ± SD, %)	(x̄ ± SD, %)		
	TP 1	TP 2	TP 3		
Tooth structure	1.98 ± 0.73 out of 3 (66.0%)	2.25 ± 0.73 out of 3 (75.0%)	2.02 ± 0.66 out of 3 (67.3%)	TP 1 vs 2 = 0.077 – 0.484	TP 1 vs 2 = 0.003*
				TP 1 vs 3 = -0.171 – 0.264	TP 1 vs 3 = 1.000
Oral hygiene	3.43 ± 0.66 out of 4 (85.8%)	3.75 ± 0.48 out of 4 (93.8%)	3.52 ± 0.60 out of 4 (88.0%)	TP 1 vs 2 = 0.163 – 0.510	TP 1 vs 2 = < 0.001*
				TP 1 vs 3 = -0.057 – 0.281	TP 1 vs 3 = 0.327
Diet	1.94 ± 0.71 out of 3 (64.7%)	2.56 ± 0.65 out of 3 (85.3%)	2.06 ± 0.67 out of 3 (68.7%)	TP 1 vs 2 = 0.416 – 0.837	TP 1 vs 2 = < 0.001*
				TP 1 vs 3 = -0.069 – 0.312	TP 1 vs 3 = 0.370
Common oral diseases	2.13 ± 0.83 out of 3 (71.0%)	2.68 ± 0.61 out of 3 (89.3%)	2.39 ± 0.82 out of 3 (79.7%)	TP 1 vs 2 = 0.342 – 0.742	TP 1 vs 2 = < 0.001*
				TP 1 vs 3 = 0.28 – 0.495	TP 1 vs 3 = 0.023*
Total	9.47 ± 1.69 out of 13 (72.8%)	11.24 ± 1.47 out of 13 (86.5%)	9.99 ±1.68 out of 13 (76.8%)	TP 1 vs 2 = 1.324 – 2.213	TP 1 vs 2 = < 0.001*
				TP 1 vs 3 = 0.083 – 0.954	TP 1 vs 3 = 0.014*

A repeated-measures ANOVA with a Greenhouse-Geisser correction indicated that the scores differed significantly over time (F(1.857, 198.671) = 57.512, p < 0.01). A post hoc analysis with a Bonferroni adjustment showed that the knowledge score significantly increased from baseline to immediately after the intervention (+1.77 points, p < 0.001) and from baseline to three months after the intervention (+0.52 points, p = 0.014), as shown in Table 4. Children also significantly increased their knowledge from baseline to after three months in tooth anatomy, food concerns, brushing techniques, and oral pathologies. At each time point, the results did not statistically differ between groups and did not vary with gender, age, or access to a dentist/oral hygienist.

## Discussion

Although positive trends were noted in oral hygiene behaviours, these changes did not reach statistical significance, likely due to relatively high baseline values and a ceiling effect (93.5% brush their teeth daily, 88.9% brush more than two times a day, 42.6% floss daily, and only 8.3% did not attend the dentist every year). During calculations to obtain the required sample size, we assumed our population would follow the previously documented national panorama, with approximately 70% of children brushing their teeth daily. However, we found that this starting point was significantly higher than expected (> 90%). Therefore, although our sample size was adequate, it did not have statistical power due to a ceiling effect. We highlight that, when compared to national values, our sample had better baseline behaviours concerning tooth brushing but not tooth flossing (e.g., brushing their teeth daily - 93.5% vs. 69.9%; flossing daily - 42.6% vs. 68.3%).

On the other hand, our data suggests a gain in knowledge post-intervention, with an average increase of 1.77 points in the oral health knowledge score, and partial retention three months later (+0.52 points). This finding indicates that the educational material was effective in conveying content and capturing the children's attention, corroborating the effectiveness of visual and interactive pedagogical methods in promoting health knowledge among children, as described in previous research [12, 13]. Knowledge gains in subtopics such as dental anatomy, nutrition, brushing techniques, and oral diseases reinforce the relevance and appropriateness of the video content for the target age group. However, the slight decrease in knowledge after three months highlights the need for continuous and systematic reinforcement strategies, such as integrating this content into the school curriculum or periodically repeating educational sessions.

Although the individual use of verbal explanations, animations, and videos is equally effective in providing children with relevant information about oral hygiene, some literature suggests that animation videos may be more favourable, accessible, and consumable for the newer generations [14]. The use of educational videos, pedagogically adapted to the children's age group, is nowadays a feasible, reproducible, and effective strategy in the majority of school settings. Moreover, the use of video-based materials presents a significant advantage in terms of content standardisation, ensuring consistent delivery of information across diverse contexts, namely in schools. This uniformity may contribute to the equitable implementation of the intervention and facilitate its adaptation and dissemination in future research and practice [11, 15-17].

This video-based educational intervention proved effective in promoting immediate improvement in oral health knowledge among school-aged children. However, the translation of this knowledge into consistent and appropriate hygiene practices remained limited. These findings emphasise the need for integrated, multicomponent, and sustained strategies to achieve meaningful and lasting improvements in children's oral health outcomes.

Study limitations

Our study faced several limitations, including reliance on self-reported data from children and the absence of a control group. Contextual variables, such as parental involvement, family health knowledge levels, and access to health resources, might strongly influence behaviours. These were not explored in this study and are an essential component of the oral health-related behaviours.

Several aspects of this study related to the participants' characteristics, namely school absenteeism, might have conditioned questionnaires’ completion and influenced the drop-out rate. To ensure maximal consistency in the answers and to reduce missing data, the authors only included children who completed all questionnaires. Moreover, this study was entirely based on self-reported data with no input or correction from schoolteachers or parents, which could also have influenced the given answers regarding knowledge and habits - this factor might be the main factor that explains why, although the DMFT was so high in these two schools, the knowledge scores were also very high from the beginning. In future works, children's habits and knowledge should be compared with their baseline oral health (e.g., the number of cavities at the time of our intervention) and with a formal examination on how they brush/floss their teeth.

For future works, the authors believe that the inclusion of randomised control groups might also enable a more robust evaluation of the intervention’s effectiveness and possibly establish causal inferences. Moreover, using clinical assessments to complement questionnaire-based data could also offer a more comprehensive analysis of the intervention's impact on children’s oral health outcomes. Additionally, the assessment of psychosocial indicators or parental involvement may contribute to a better understanding of the underlying factors influencing behavioural change.

Whenever possible, interventions and teaching classes should consider the integration of not only video-based resources but also complementary tools such as real-life demonstrations or anatomical models. These additions may enhance the clarity and comprehensibility of the information conveyed, facilitating greater student engagement and improving the overall effectiveness of the educational sessions.

## Conclusions

As behaviour modification is a gradual process, extended interventions incorporating both family reinforcement and professional support are more likely to yield consistent and lasting improvements in oral health behaviours. This video-based intervention enhanced student engagement with oral health content, offering valuable insights into how such approaches can improve interest and engagement. It also highlighted the potential of digital education in promoting better oral health knowledge among children.

## Figures and Tables

**Video 1:** Happy teeth episode 1: dentition, structure and function of teeth

**Video 2:** Happy teeth episode 2: food and oral health

**Video 3:** Happy teeth episode 3: dental hygiene techniques

**Video 4:** Happy teeth episode 4: diseases of the teeth and gums

**Figure 2:**
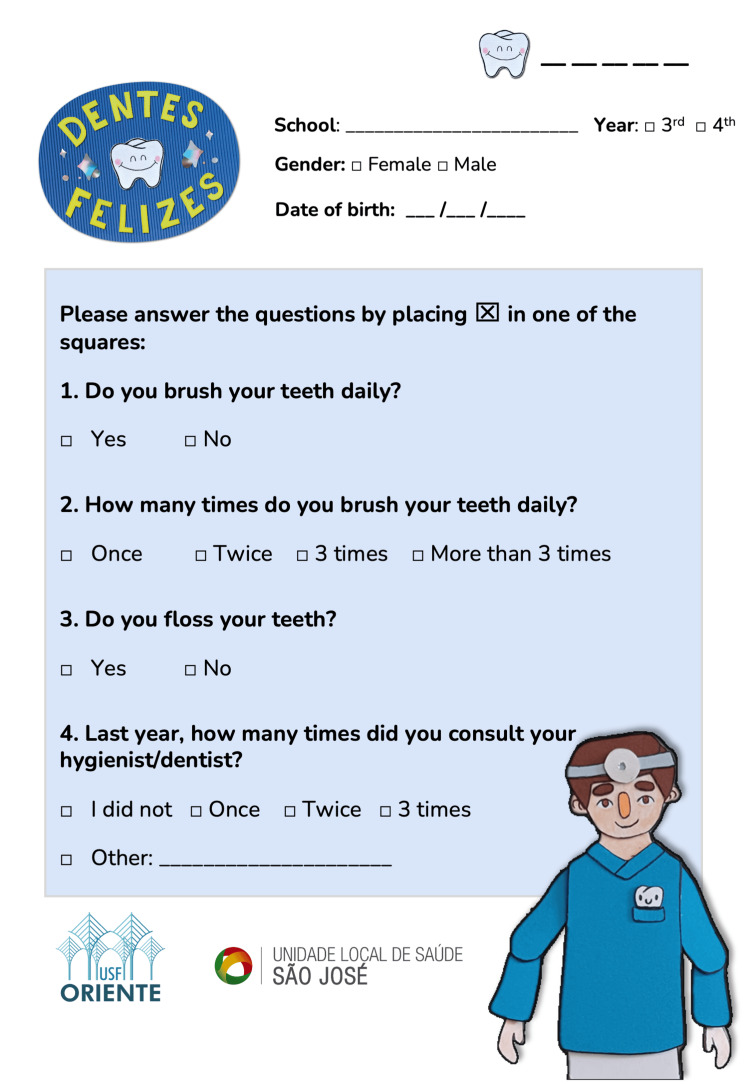
Behaviour characterization questionnaire

**Figure 3:**
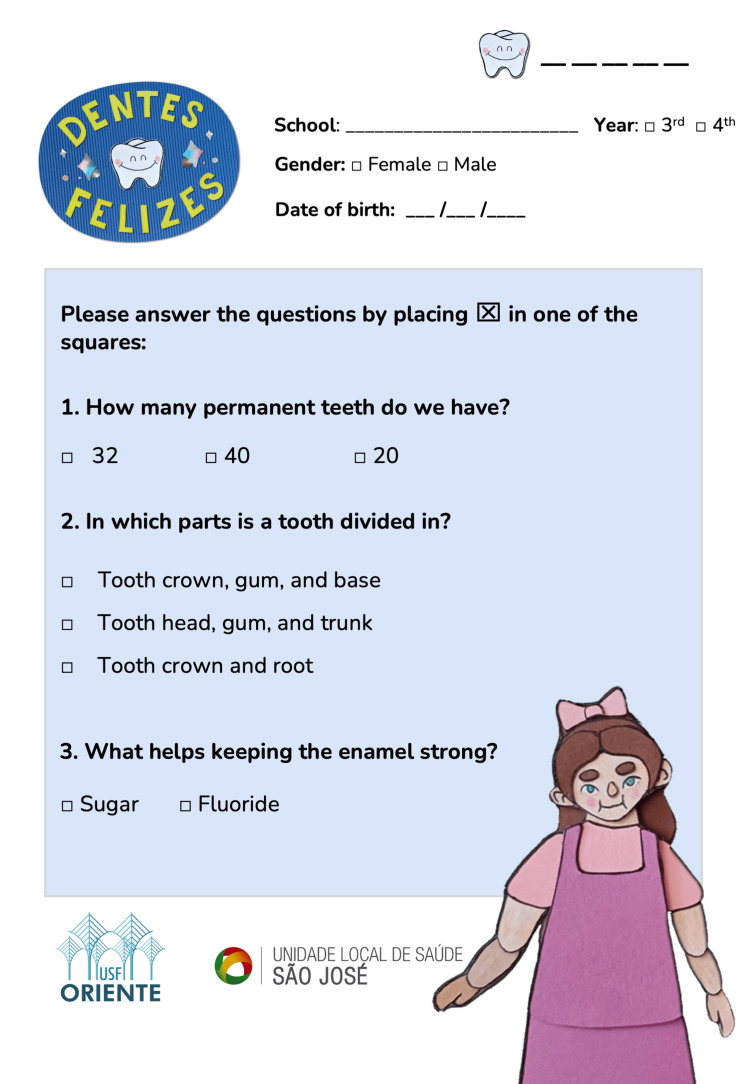
Tooth structure questionnaire

**Figure 4:**
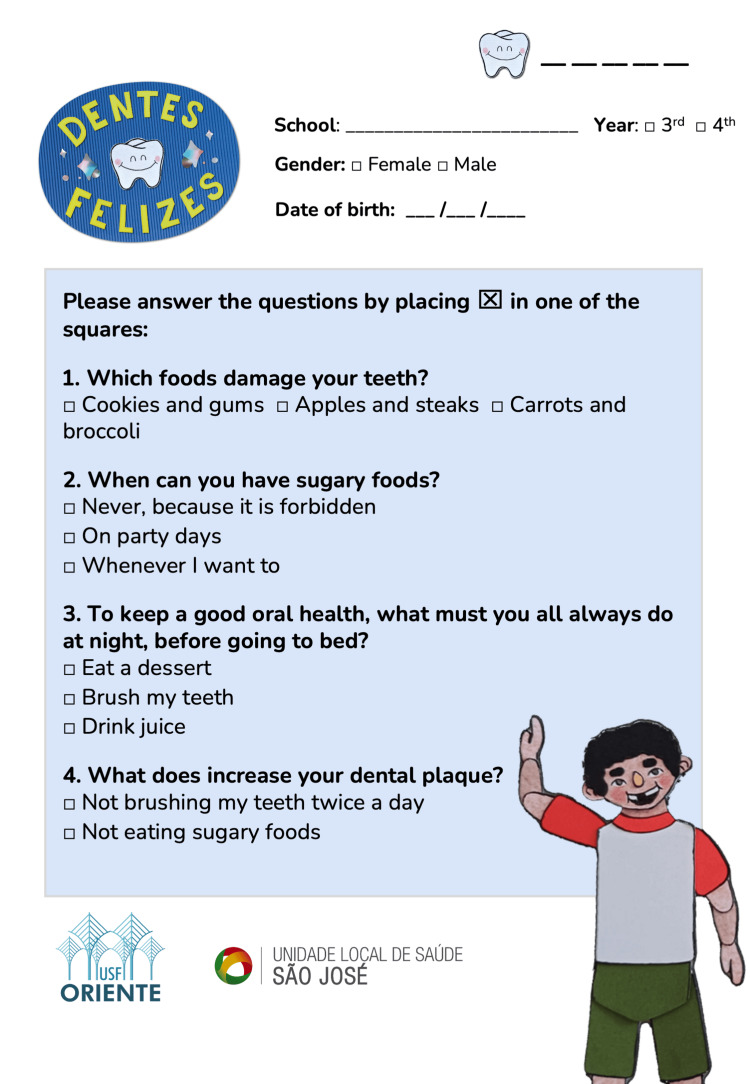
Diet questionnaire

**Figure 5:**
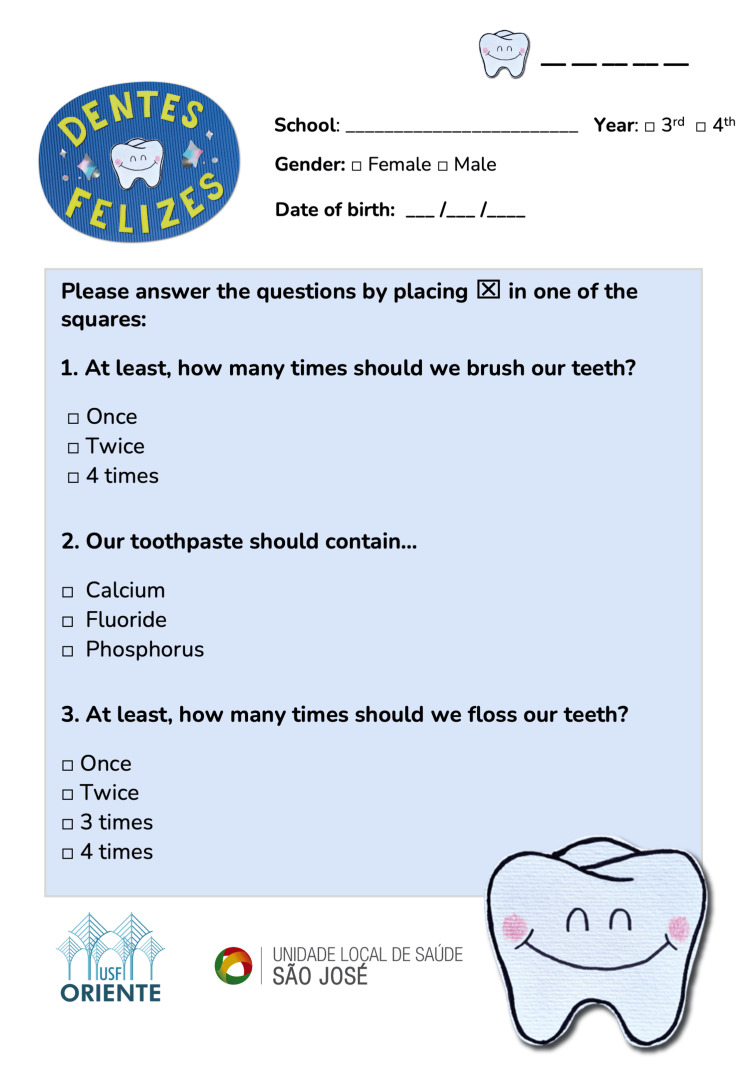
Oral hygiene questionnaire

**Figure 6:**
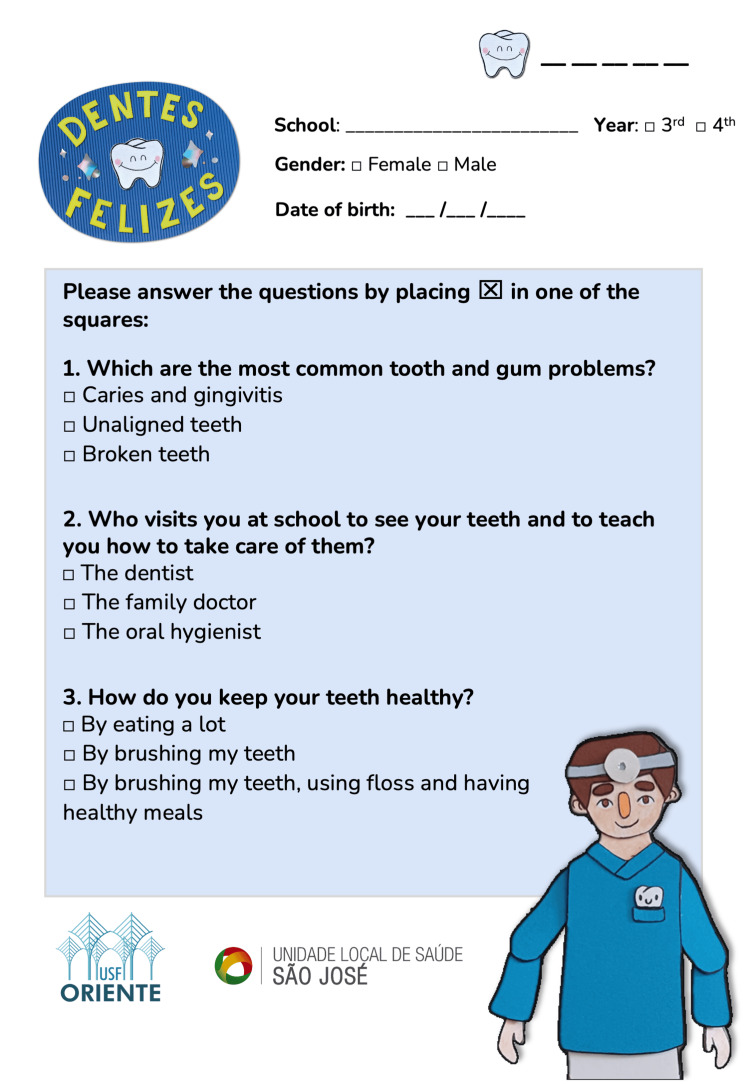
Common oral diseases questionnaire

## Appendices

Appendix 1 

Appendix 2 

 Appendix 3

Appendix 4 

Appendix 5

Appendix 6 

Appendix 7 

Appendix 8 

Appendix 9 
